# American Proctologic Society

**Published:** 1907-08

**Authors:** 


For Texas Medical Journal.

### American Proctologic Society.

NINTH ANNUAL MEETING, HELD AT ATLANTIC CITY, N. J., JUNE 3 AND 4, 1907.

---

The President, Dr. Samuel G. Gant, in the chair.

### OFFICERS ELECTED.

The following officers were elected: President, A. Bennett Cooke, M. D., Nashville, Tenn.; Vice-President, Louis J. Krouse, M. D., Cincinnati, Ohio; Secretary-Treasurer, Lewis H. Adler, Jr., M. D., Philadelphia, Pa., and the Executive Council, J. Rawson Pennington, M. D., Chicago, Ill., Chairman; Samuel G. Gant, M. D., New York City, N. Y.; A. Bennett Cooke, M. D., Nashville, Tenn.; Lewis H. Adler, Jr., Philadelphia, Pa.

The place of meeting for 1908 is Chicago, Ill., the time to be announced later.

### ELECTION OF MEMBERS.

The following were elected members of the society: Dr. Jerome M. Lynch, of New York City; Dr. Jas. A. McVeigh and Dr. J. A. MacMillan, of Detroit, Mich.

The following is an abstract of the principal papers read:

### PRESIDENT'S ADDRESS.

The President, Dr. Samuel G. Gant, of New York City, said "that the annual meetings of the Society were like a post graduate school where advanced information in proctology could be obtained by the members. He considered it unwise to admit to membership in the society the general surgeon and the young rectal specialists of less than five years' experience in this special work, because the membership would become too large and the papers contributed by them would not meet the requirements.

He maintained that the proctologist of the future, in order to be successful, must have a thorough literary and medical education, a hospital training and clinical facilities, and that he must be clever, industrious and persistent. He emphasized the necessity of educating both the profession and the laity as to the remarkably improved methods now employed in the handling of patients suffering from disease in the lower bowels. He also said it was the duty of the proctologist to demonstrate by his work and writing that most tubercular fistulæ were curable and that the curing of ordinary fistulæ did not tend to bring about lung or skin affections, as was formerly believed; that fecal incontinence does not follow fistula operations when the muscle is cut at a right angle and the wound is properly dressed; that many rectal diseases, such as fissures, ulcers, small fistulæ and some hemorrhoids can be operated upon under local anesthesia, and that in the majority of instances constipation and chronic diarrhea are curable by local and surgical measures.

Finally, he emphasized the fact that the etiology of many discomforts, nervous and reflex phenomena, usually attributed to the genital organs is frequently to be found in some pathologic process located in the sigmoid rectum or anus."

### REPORT ON PROCTOLOGIC LITERATURE FROM JUNE, 1906, TO JUNE, 1907.

Dr. Samuel T. Earle, Baltimore, Md., read his report on Proctologic Literature, covering a period from June, 1906, to June 1907, in which he said "That, while there has been nothing startling in proctologic literature in the past twelve months, your committee is gratified with the steady progress in this branch of medicine and surgery, as has been reflected in the literature on this subject. Especially gratifying have been the recommendations for the radical treatment of carcinoma of the upper rectum and sigmoid, as set forth in the papers of Samuel G. Gant, W. J. and C. H. Mayo, and James P. Tuttle, in which they all recount the combined advantages of the abdominal and perineal routes, which we think will greatly lessen the likelihood of recurrences, and increase the number of permanent cures. We note with pleasure the systematic efforts that are being made in the study of the etiology of pruritus ani. The paper of Wallace of London in 1905 on the study of this question stimulated the efforts of others in this direction, and we find an excellent paper by J. C. Hill, of Boston, on the same, in the *Boston Medical and Surgical Journal,* 1906. In this article he takes the stand with Wallace that there is always a cause for this malady is which the pruritis is only a local symptom. We quote him as follows: "Pruritus ani is the symptom caused by unnatural moisture or discharges, produced either by lesions about the anus or by congestion, or some pathologic condition in the rectum or sigmoid. It is due to one of five causes. First, and by far the most important, are superficial ulcerations, or abrasions of the anal canal. Second, catarrhal diseases. Third, external hemorrhoids. Fourth, inflammation or irritation of the crypts of Morgagni. The free borders or valves of these crypts consist chiefly of nerve fibres, ganglion cells and connective tissue. When inflamed or infected, they may give rise reflexly to pruritus ani. The writer calls attention to the tits which project from the margin of these valves as accessory sense organs. When hypertrophied or elongated they cause many distressing symptoms about the anus, viz., creeping, crawling sensations and itching. Fifth, small polypi of the anal canal."

It is not only necessary to remove the exciting cause of the disease, but also to direct appropriate treatment to the unnatural conditions of the skin. In this connection your committee is glad to be able to report a number of cases of the aggravated form of this disease which had failed to respond to the usual local and constitutional methods that were successfully treated by Dr. Ball's recommendation of dividing the anal nerves. These have been reported by Drs. Mattin and Earle.

Dr. Charles B. Kelsey calls attention to the office treatment of hemorrhoids by puncture with the electric cautery. The method is to make numerous punctures with a pointed cautery to the internal hemorrhoids. This method has been used for the past ten years in lieu of that by injection, with most satisfactory results, and without any unfavorable effect. For the details of the method I would refer to his article in the *Therapeutic Gazette* for March 15, 1906. The reviewer would respectfully report with reference to this suggestion that about ten years ago he tried in a number of cases, but with only temporary success where the hemorrhoids were large. It answered very well in small capillary hemorrhoids. J.

A. Hartwell in the *Annals of Surgery,* Philadelphia, for 1906, Vol. 43, page 146, reports a case of resection of the rectum for syphilitic stricture, with end to end anastomosis. As might have been predicted, it recurred, and while it was necessary to continue dilating, he was not hopeful of results. Your committee would remind its members that such radical measures seldom accomplish the desired results, as recurrence is almost inevitable.

"ADDITIONAL EXPERIENCE WITH A NEW PROCEDURE IN OPERATING FOR ANO-RECTAL FISTULA.

By Dr. J. R. Pennington, Chicago, Ill., who said: "I have found the employment of the seton in operating on cases of ano-rectal fistula greatly aids in preserving the contour of the anus and the functions of the sphincter muscles."

The technique in using the seton is as follows: After all of the fistulous tracts external to the sphincter are divided a probe-pointed director is passed into the bowel through the remaining tract and an incision made on its distal side. This incision should extend far enough distally to divide all or a part of the fibers of the external sphincter and in such a direction as to locate the transferred internal opening at or near the anal margin. Then, turning the knife, make an incision—Salmon's "back-cut"—on the proximal side of the tract. A seton is then passed through the opening entering the bowel and tied loosely around the tissues remaining and undivided.

The wound is dressed as after the ordinary incision operation for fistula. At the end of twenty-four to thirty-six hours the wound is redressed, care being taken to dress it so that the opening entering the bowel will be made to heal from the proximal toward the distal side. The object in doing this is to advance the final fistulous tract as far distally (toward the skin) as the case will permit, so that, if possible, it will pass through or distally to the fibers of the external sphincter, when the healing process is complete.

As a rule the enlarged tract entering the bowel soon closes, with the exception of the part through which the seton passes. As soon as this has occurred the seton may be removed, and by the time the external wound is healed the tract entering the bowel will possibly have closed also. Should it not, at any time later this little tract may be dissected out and the remaining fibers of the muscle sewed together, thus preserving the contour of the anus and the functions of sphincters.

"OCCULT HEMORRHOIDS FROM THE RECTUM."

By Dr. William H. Beach, Pittsburg, Pa., who stated:

1. Occult blood in the stool indicates disease high up in the gastro-intestinal tract.

2. Accompanied by certain rational symptoms, as pain localized, the origin of occult blood can be noted.

3. The discovery of blood in the stool may enable us to predict hemorrhage or prevent disaster.

4. The most frequent sources of occult blood are in the order named: Stomach, duodenum and caput coli.

5. The aloin-turpentine test, as practiced by Dr. J. Dutton Steele, of Philadelphia, is recommended.

6. Proctologists should make an examination for occult blood a routine practice in cases of anemia accompanied by diarrhea or constipation.

"ETIOLOGY AND SYMPTOMS OF FISSURE."

By Dr. C. F. Martin, of Philadelphia, Pa., who said: "The fissure is usually situated posteriorly and directly over the 'white line' of Hilton, due to the arrangement of the fibers of the external sphincter and to the fact that the anal canal has less elacticity directly over that line. Most fissures start as the result of the distention of the canal by hard feces and excessive straining at stool. Those situated anteriorly, are often seen after confinement due to the pressure of the fetal head upon the perineum.

The sentinel pile is noticed in most cases if the fissure has been present for any length of time. It is a simple inflammatory hypertrophy. Hypertrophy of the anal papillæ does not appear to be an important factor in the causation of fissure. True hypertrophy of the sphincters is rarely seen, but in its place we find an excessive irritability of the external sphincter.

The distinctive symptom of this disease is pain or sphincteralgia, preceded by a "pain interval" of from one minute to an hour, during which time the patient has comparative comfort. The pain is caused by spasm of the sphincter compressing the nerves in the ulcer. This contraction interferes with the perianal circulation and renders the inflamed nerves more sensitive. The "pain interval" is caused by a temporary improvement in the perianal circulation produced by the straining efforts at stool.

The constipation of fissure often precedes the formation of the ulcer, an irritable sphincter being the underlying factor in the production of this condition. The constipation increases after the formation of the ulcer due to the fear of the patient of the pain following stool.

The treatment consists in the divulsion of the sphincters and the usual stimulating after treatment. The sentinel pile should be removed at the time of divulsion. Division of the external sphincter is not advised, for frequently it does not unite and eventually atrophies. Fistulæ, abscesses and fecal impaction are mentioned as frequent complications of fissure and call for appropriate treatment.

"LOCAL VS. GENERAL ANESTHESIA IN RECTAL SURGERY."

By Dr. G. B. Evans, Dayton, Ohio, who stated that "Pain naturally is the common curse and dread though relatively essential of the human family.

"The law of self-preservation when an individual is threatened with pain is at once a law of resistance, manifest by intense expectancy and defiant attitude.

"The shock incident to the terror of pain is incomparable to that which is likely to follow an abbreviated use of a general anesthetic. In consideration of the evolutionary plane occupied by the average American of today and the more remote period of his removal from the gorilla peripherial sensibility of the jungle, we are forced to conclude that he is more sensitive and in need of greater consideration for the relief of pain. Because an operation can be done painlessly, it does not follow that there will not be subsequent suffering and some, and perhaps severe, shock.

"But in this nerve block period of Creile and Pennington, and the Gant period of dermal and sub-dermal distension, we are told there is scarcely any use any more for a general anesthetic. Do we not believe as proctologists that in our operative field sensibility is most difficult to abolish, and would it not naturally appear that more narcosis is necessary in this kind of surgical work and as a consequence more shock? Again, would it not be possible by the combined use of general and local anesthesia less shock would ensue—and more operations could be made with success. I believe that by using the local anesthetic preceding the general anesthesia we lessen the amount of the general anesthetic appreciably, diminish the dread and fear, and consequently diminish shock and danger thereby. Therefore, the combined method of narcosis, less anesthetic, suspending shock incident to conscious dread, as well as anesthetic shock, rendering more complete operative area—consequently more satisfactory work. It is simple to operate upon prolapsing piles, but not so simple to operate upon piles above the sphincters yet demanding operative interference.

"The injections of sterile solutions disturbs the parts anatomically. The frequent punctures through the tissues invite infection, and infection trouble. You can never measure the nerve and seldom the surgical all of your patient. Its chief advantage is that occasionally you are able to do work in your office and even then unsatisfactorily.

"I fail to see the advantage of operation under local anesthesia in your office and follow your patient home and give hypodermic or morphia over the usual custom of operating at home in the first place.

"An operation for hemorrhoids is a matter of some seriousness —often attended by some shock—often bloody, and the greatest caution should be observed that asepsis be obtained.

"I do not think it best for our patients that general practitioners should be taught that aseptic precautions at the time of operation and rest at home for a few days is unnecessary. Do we not magnify the applicability of local anesthesia, will not accidents occur, are we not sacrificing perfect scientific work, are we not belittling our chosen work—a class of operations that are important and not free from danger?"

"THE SIGMOIDAL FACTOR IN CONSTIPATION."

Dr. E. A. Hamilton, Columbus, Ohio, said: "In a certain per cent of intractable cases of constipation, organic change in the wall of the sigmoid is the controlling factor. This change is entirely independent of any disturbance which may occur on the outer surface of the viscus; *e. g.*, malposition and pathologic flexures due to adhesions. The change in the gut occurs in the sub-mucous and muscular coats and consists of a round cell infiltration of these coats, which subsequently contracts, thereby to a greater or less degree narrowing the lument of the bowel. The round cells change into spindle cells subsequently undergoing a metamorphosis into true connective tissue. The contraction of this connective tissue so narrows the caliber of the sigmoid that constipation of an obstinate type must result. This change is not always limited to the sigmoid, it frequently involves the descending colon as well; in addition to the contraction of the gut it loses also its resilience, which further adds to the difficulty of the passage of fecal debris. The etiologic factor is the absorption of bacteria and toxic products from the sigmoidal contents. The mesentery is also involved, and is thickened and shortened. The whole process is chronic, several years being required to bring on the condition. Surgery offers the only relief. An anastomosis must be effected by any suitable surgical procedure between the unaffected position of the intestinal tract above and below the lesion.

"A REPORT OF TWO CASES OF SIGMOIDOPEXY."

Dr. S. T. Earle, Baltimore, Md., who said: "That in one of the cases there was the third degree of prolapse of the rectum, the invagination of the upper part into the lower portion of the rectum; in the other case there was a very acute flexure of the sigmoid upon the rectum, both of the conditions, as is well known, are frequently due to an abnormally long meso-sigmoid. The symptoms in each case were obstinate and persistent constipation, frequent bearing down pains in the lower pelvis, a sense of weight and especially a feeling of unrelief for some hours following a stool, or an attempt at the same; associated with these local symptoms were darting pains in various parts of the body, nausea, anorexia, frequent headaches and the various neurotic symptoms that go to make up a typical case of neurasthenia. The case of invagination was diagnosed positively by a digital examination while straining at stool, the sulcus being distinctly felt with the finger; the case of acute flexure was diagnosed by means of the protoscope, the flexure being so acute that it was only possible to enter the sigmoid with the protoscope by getting the end of the latter around the flexure and pulling it aside. The flexure was so acute that it obliterated the lumen of the bowel at this point. The technique of the operation is such as is given in Tuttle & Gant's works on *Diseases of the Rectum and Anus.* I met with no special difficulty in performing the operations. The meso-sigmoid was very long in both. I was particular in pulling off the abdominal peritoneum where the sigmoid was to be held in apposition and also in attaching the sigmoid to the transversalis fascia. Both cases made good recoveries, except that one was retarded by a stitch abscess. The results in both cases were most satisfactory and pronounced, with almost immediate relief of the persistent and obstinate constipation, with the gradual disappearance of the neurotic symptoms. Dr. Gant has recently reported a number of sigmoidopexies and colopexies with most satisfactory results.

"Dr. Clark, in a paper read before the Medical and Gynecological Section of the Medical and Chirurgical Faculty of Maryland, on February 15, 1907, called attention to the frequent associations of gastroptosis, floating kidney and enteroptosis in the same individual."

"FECAL IMPACTION."

Dr. Lewis H. Adler, Jr., of Philadelphia, Pa., in a paper entitled "Fecal Impaction," called attention to the result of obstipation, or an attack of constipation causing an accumulation of feces in the cæcum or in any part of the colon; but the term impaction, the subject of the paper, should be usually employed when such an accumulation occurs in the pouch or ampulla of the rectum, or in the sigmoid flexure.

Attention was called to the difference between an ordinary persistent constipation and an impaction, as the latter may follow from a single attack of constipation; whereas obstinate constipation may never, or only after a long period, cause impaction. The symptoms of the two conditions are also very different, as an impaction is usually marked by a diarrhoea, whereas chronic constipation is associated with costiveness. After calling attention to the various causes of the malady under consideration and the symptoms of the same, the treatment was detailed as consisting primarily in the removal of the mass, and secondarily in the relief of the inflammation of the mucous membrane occasioned by the irritating presence of the fecal matter as well as the removal of all causes which contribute to the constipated habit, which is undoubtedly the prime factor in most cases in producing an impaction.

The easiest manner of breaking up the fecal mass is to put the patient under an anesthetic and then to forcibly divulse the sphincters, after which the mass may be disintegrated by means of the finger, a lithotomy scoop or an old-fashioned iron spoon. In women considerable assistance may be rendered by passing a couple of fingers into the vagina, and by this means steadying the mass so that it may be the more readily broken.

In some instances the writer was able to break up an impaction without resorting to anesthesia, simply by using the finger and sometimes by the additional use of a bivalve speculum and a rectal scoop or spoon.

Previous to resorting to instrumental aid in the removal of the impaction, the fecal mass may be softened and its passage facilitated by the use of enemas—especially is this so in cases in which the sigmoid is the part affected—in which situation material assistance can not be gained by the employment of instruments. For the purpose of administering the injection a douche-bag holding several quarts is to be preferred. The injection substance should be composed of soap and water, to which I have found the addition of glycerine of considerable benefit, a dessert-spoonful to a quart. When the impaction is in the sigmoid, the injection should be given through a Wales bougie, preferably the one modified by Dr. Dwight H. Murray, of Syracuse, New York, which is stiffer than the ordinary article sold, which latter is frequently useless for the purpose intended, as it readily doubles on itself, and a high injection is rendered impossible by its use. When this method is employed the patient should be placed in the knee-chest posture.

A word of caution should be given here as to the danger surrounding the unguarded use of drastic purgative drugs in cases of impaction. By their employment peristalsis is increased and the fecal mass softened, but the bowel in its inflamed and distended condition may be thereby the more easily ruptured, and, if in addition a stricture is present, the caliber of the gut may be entirely occluded by forcing into it the hard fecal mass with the attendant symptoms and consequences of total obstruction of the intestines. So much for the treatment of the actual impaction.

"PRURITIS ANI—IS IT A DISEASE PER SE OR MERELY A SYMPTOM?"

Dr. Louis J. Krouse, Cincinnati, Ohio, who quoted from the works of Bodenhamer, Agnew, Wright, Ball, Crepps, Gant, Matthews, Tuttle and others, their opinions regarding the etiology of this disease, and then stated, "Pruritus ani essentials is a disease which is due not to a local but to a constitutional cause, and is due to some trophic changes in the nerves supplying the parts." He further stated that the changes occurring in the skin of the anus and surrounding parts, namely, the hypertrophy, the loss of pliability, and the absence of pigment, can only be explained on the faulty nervous supply of the parts. He showed that an increase of pigment ought to accompany severe itching, and not a total disappearance, and finishes his article by saying that "The absorption of the normal coloring matter of the affected area does occur, notwithstanding that the epidermis was not destroyed," and said "A similar process of absorption takes place in lencoderma. All authorities acknowledge that the cause of the latter disease is to be found in the nervous system," and concluded with the statement "That pruritus ani, at least in such cases, is a disease *per se*, and not a symptom."

(a) Dr. Dwight H. Murray, of Syracuse, New York, presented a new Hemorrhoidal Clamp, which had the following qualities in combination, that make a first-class instrument, viz., scissors-shaped, parallel jaws, can be closed and released instantly without the use of a thumb screw, thereby saving much time while operating.

The Goodell dilator reversed is used as the ground principle for the lock.

(b) Dr. Dwight H. Murray, of Syracuse, New York, reported the case of a man 48 years old, who had been troubled with sciatica in the right leg for two years, and had also been a sufferer from hemorrhoids for ten years, having frequent profuse hemorrhages therefrom.

The hemorrhoids had not been treated. The sciatic nerve had been stretched and treated by various methods by a physician at his home town, included in which was the following, completed in three sittings two days apart.

At the first sitting, six hypodermic injections 1-150 gr. of atropine each were given into the sheath of the sciatic nerve. At the second sitting, seven injections of the same amount, and at the third sitting, eight injections were given as before and one extra into the nerve before it leaves the pelvis. The patient was unconscious for fourteen hours after the last sitting, very little improvement resulted.

In November, 1906, the author was first consulted, and on December 12, 1906, operated on him for internal hemorrhoids. He made the usual recovery up to the ninth day, when there was a sudden profuse secondary hemorrhage. The patient was almost exsanguinated before the author arrived.

He immediately examined, found the superior hemorrhoidal artery was throwing a full-sized stream; this was secured, the patient stimulated and made an uneventful but slow recovery. The patient has had no sciatic pain since the operation.

Dr. Murray concluded that inasmuch as sciatica is often symptomatic, that no such severe treatment is justified until all possible reflex causes are first removed.

The cause of the hemorrhage was probably due to the thrombus or eschar at the end of the vessel being thrown off before thorough healing had taken place, and was influenced largely by his general anemic condition before operation.

"THE TREATMENT OF ISCHIO-RECTAL AND PELVI-RECTAL ABSCESSES."

Dr. T. Chittenden Hill, of Boston, Mass., who said "That he employed general anesthesia produced with ethyl chloride for the ischio-rectal abscess and ether anesthesia for the pelvi-rectal abscess.

His experience with infiltration anesthesia has been unsatisfactory. He emphasized the importance of an early incision for peri-rectal abscesses, claiming that when acute symptoms have existed for a day or two, with pain and tenderness, even before there is much edema or discoloration of the skin, long before fluctuation can be detected, an incision may prevent abscess formation by allowing the escape of blood or serous exudate from the engorged blood-vessels.

He advised a T incision and breaking up the existing septa with the finger, after which the sphincters are divulsed. He believed squeezing, scraping or disinfecting an acute abscess to be a great mistake, as it only serves to destroy the new granulation tissue and to spread the infecting bacteria. For the deeper ischio-rectal and in all pelvi-rectal abscesses he recommended rubber drainage tubes, discarding their use as quickly as possible in the after-treatment.

He believed that the great majority of pelvi-rectal abscesses should be reached by perineal dissection."

"CRYPTITIS."

Dr. J. Coles Brick, Philadelphia, Pa., who said: "The anal valves and crypts, first pointed out by Morgagni, and called after his name, are found as vestigical remains of the junction of the rectal mucous membrane with the skin. They vary in number and size, but are absent in the anterior and posterior commissures. They have no known functions, but are the cause of obscure symptoms, when diseased, and from the fact that the valve or covering part of the crypt may conceal the diseased area, repeated examination will fail to show the lesion, unless each crypt is probed, when tenderness or pain will be felt. A conical fenestrated speculum is the best to use, and when the diagnosis has been made the valve should be removed and the crypt converted into a raw surface, so that healing will obliterate it."

Dr. A. B. Cooke read a paper on "Observations on Certain Points in the Anatomy and Physiology of the Rectum." He expressed the view that the usual conception of the external sphincter muscle is erroneous; that under normal conditions it is not in a state of tonic contraction, but, on the other hand, is at rest and passive, the shape of the muscle and the arrangement of its fibres being such that the anal aperture is maintained in a state of passive closure. It is not conceivable that a voluntary muscle should require the constant action of nerve force to keep it in a state of rest. The only action of this muscle is to voluntarily oppose or terminate the act of defecation by tonic contraction.

With reference to the internal sphincter, the essayist observed that there is no occasion to credit this muscle with any special action in addition to that of the circular coat of the bowel, of which it is a part. By reason of its location and thickness, it probably exercises some passive sphincter control, but its chief action is undoubtedly that of a detrusor, serving to complete the expulsion of feces and keep the anal canal free of contents.

The levator ani muscles, acting together, constitute the sphincter of the proximal extremity of the anal canal. To understand this it is only necessary to remember (1) that the upper or pelvic surface of these muscles presents a deep, funnel-shaped concavity, the beginning of the anal canal being at the lowest point; (2) the strong bundles of fibres which unite immediately behind the rectum arise in front from the pubis and anterior portion of the fascial line and pass downward and backward in close relation with the lateral walls of the rectum, crossing it obliquely at the upper limit of the anal canal.

The well known difficulty of voiding urine while a costive stool is being expelled, which is usually attributed to the action of the levatores ani, is due rather to the pressure of the fecal mass upon the prostatic and membranous portions of the urethra, since, at the time of defecation, these muscles, like the sphincters, must be in a state of relaxation.

The part played by the anal canal in defecation is purely passive, except at the completion of the act when the voluntary muscles which enclose it are strongly contracted, expelling any remnant of feces and bringing its walls again into their normal relation when at rest of close apposition.

The essayist dissented from the commonly accepted teaching that there is an inhibitory center in the cord which presides over the action of the external sphincter and which is called into action at the time of defecation to inhibit its tonic.

The relaxation which occurs at such times seemed to him fully explained by the mechanical pressure of the descending mass upon a structure which only offers passive resistance unless contracted by voluntary effort, and which possesses sufficient resilience, independent of any nerve influence, to regain its normal form and tone as soon as the pressure is removed.

